# COVID-19 Vaccines in the Pipeline, Are Antibodies Adequate?

**DOI:** 10.3390/vaccines9030241

**Published:** 2021-03-10

**Authors:** Md Kamal Hossain, Majid Hassanzadeganroudsari, Jack Feehan, Vasso Apostolopoulos

**Affiliations:** 1 Institute for Health and Sport, Victoria University, Melbourne, VIC 3011, Australia; md.hossain18@live.vu.edu.au (M.K.H.); majid.hassanzadeganroudsari@vu.edu.au (M.H.); jack.feehan@vu.edu.au (J.F.); 2 The Department of Medicine-Western Health, The University of Melbourne VIC, Melbourne, VIC 3021, Australia

The COVID-19 pandemic caused by the novel coronavirus strain SARS-CoV-2 has already led to catastrophic consequences in global physical and psychological health, as well as economic recession [1] and its advance is ongoing [2]. Since its declaration by the World Health Organization as a pandemic on 11 March 2020, there have been over 2.5 million deaths from more than 111 million infections worldwide [3]. Researchers are working at a feverish pace to develop COVID-19 vaccines to halt the spread of SARS-CoV-2 and limit the associated fatalities. A significant body of data has already been produced, mostly from phase I trials, and a smaller number of phase II and III trials. However, the data produced has been particularly focused on the production of neutralizing antibodies as a proxy for efficacy; however these studies cannot predict the long-term preventative capacity. This is partially because the main focus of the trials was safety, and dose adjustment as well as to gain an indication of immune response in small numbers of participants. Large scale and long-term study results are required to conclude on the overall efficacy of the developed vaccines.

The key success criteria for a vaccine is to elicit long-term antigen-specific neutralizing antibody responses by plasma cells alongside development of persistent T cells and B cell memory [4]. It has been hypothesized that to prevent severe COVID-19 infection and generate a long-lasting effect it might be necessary for a vaccine to stimulate both cellular (T cells responses) as well as humoral (antibody-based) immunities. Both are key parts of an immune response which ultimately leads to the destruction of a pathogen [5]. SARS-CoV-2 typically enters the body through the nose and throat. It then binds to and invades the cells of the upper respiratory tract which are rich in angiotensin-converting enzyme 2 (ACE2) receptor [6]; although recently it has been shown that SARS-CoV-2 can also enter host cells via several different receptors [7,8,9]. If the individual’s immune system is able to repel the virus during this initial phase (via the generation of neutralization antibodies), it is able to move down to infect the lung parenchyma, where it becomes significantly more dangerous. The epithelial linings of the respiratory tract and lung parenchyma are rich in ACE2 receptors to which SARS-CoV-2 binds through the characteristic spike protein located on its surface and invades the cell [10]. Before the virus binds and invades the host cell, B cell secreted neutralizing antibodies can bind with the spike protein, rendering it unable to infect host tissue. However, once the virus has invaded a host cell, only cytotoxic T-cell responses can kill the infected epithelial cells (Figure 1).

Several platforms are being developed for COVID-19 vaccines [11], largely focusing on the generation of ‘neutralizing antibodies’, which can neutralize viral particles making them non-infectious; however there is little emphasis on the production of active T cells that can kill infected cells and promote other immune responses, importantly including antibody production [12].

More than 150 vaccine candidates are currently in various phases of development, but a few candidates are more advanced. While these candidates have all shown the ability to generate neutralizing antibody responses, there is little known about whether they are able to generate a T-cell response. The developers released minimal data on T-cell response for evaluation (Table 1) which does not provide enough information to draw conclusion on long-term vaccine efficacy. However, until the release of full study outcomes of phase III clinical trials, consumers are unable to be sure that the responses documented in phase I and phase II are adequate to protect individuals from coronavirus infection in the long term. Surprisingly, the Russian government approved the “Sputnik V” vaccine on 11 August 2020 before Phase III trials had begun. This generated criticism from the scientific community on safety and ethics [13], as administration of vaccines without proper assessment may worsen patient outcomes when exposed to the pathogen through antibody dependent enhancement [13]. Interim phase III analysis of 9258 participants who received the Sputnik V vaccine have recently been released, and they report a favorable safety profile, and strong antibody responses [14]. They also reported an increase of interferon gamma secretion after stimulation with SARS-CoV-2 antigens, suggesting an increase in T-cell response; however this preliminary data needs further evaluation. The AstraZeneca/Oxford University vaccine Phase III trial was on halt due to unexpected unexplainable side effects and after further studies it was approved for roll-out in many countries. Recently published interim results of phase III trials of the leading vaccine candidates did not report T-cell effects, only antibody titers and preventative action again leaving questions regarding cellular immune response [15,16,17].

There are increasing concerns about the over emphasizing of neutralizing antibodies as the critical indicator of COVID-19 vaccine success [18]. In some studies, it has already been shown that patients who have recovered from COVID-19 infection demonstrate a rapid decay of antibodies against SARS-CoV-2 [18]. As such, decrease of antibody titers have been shown to be halved every 73 days, suggesting the antibodies may be depleted within a year [19]. This rate of decay is faster than previously reported for the SARS-CoV-1 virus [20]. This has raised concerns that humoral immunity against SARS-CoV-2 virus may not be sufficiently long lasting [19]. In other studies, it has been reported that patients develop a variety of immunity responses post-vaccination where some develop strong B and T cells responses, while some do not [21]. These are all alarming signals against overreliance on neutralizing antibodies as a means of achieving herd immunity or a qualification for ‘vaccination passports’. This also reinforces that T cells response is critical for the long-term protection against COVID-19 infection and these must be assessed and reported.

The generation of T-cell responses (both helper and killer T cells) has not received its due importance from vaccine developers, likely because they are comparatively difficult and very expensive to assess, particularly in large cohorts. However, a growing body of data suggests that T cells might play an important role in the success of the vaccination program to control SARS-CoV-2. If a vaccine is capable of triggering both neutralizing antibodies and T cells (CD8 T cells), it would likely provide stronger, long-lasting protection from SARS-CoV-2 virus infection. Cellular immune responses must be given appropriate attention in the analysis and reporting of COVID-19 vaccination trials, and research into vaccine platforms and adjuvants which may improve T-cell immunity is required to provide the best chance at widespread elimination of SARS-CoV-2.

## Figures and Tables

**Figure 1:**
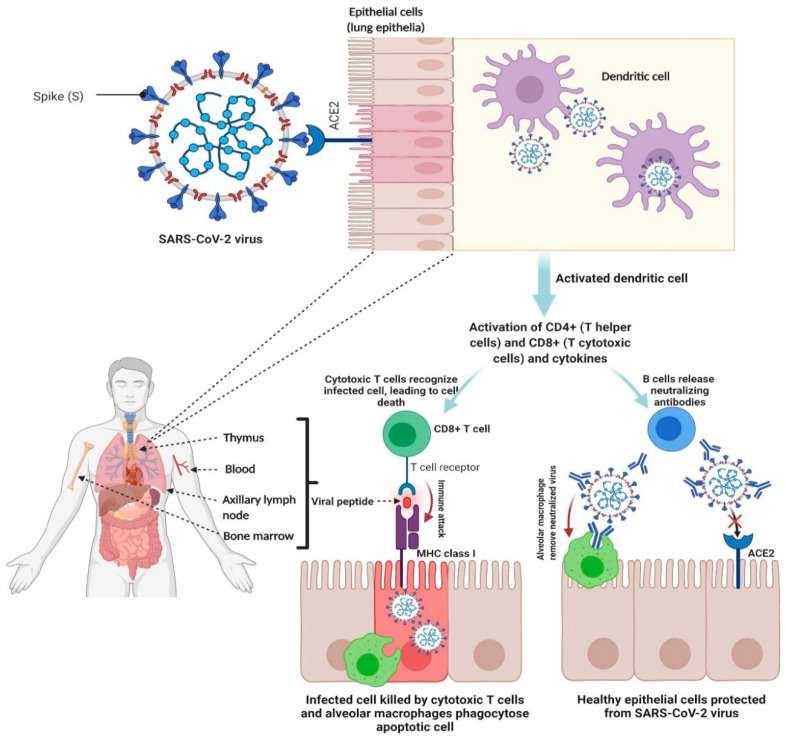
Schematic illustration of adaptive immune response in COVID-19, where B and T-cell responses are important in preventing SARS-CoV-2 infection and destroying infected cells. Figure was created with biorender.com (accessed on 9 March 2021).

**Table 1:** Summary of neutralizing antibody and T-cell response for some vaccines.

Developer and Candidate	Clinical Trial Identifier	Neutralizing Antibodies	T Cells Responses
Oxford University/AstraZeneca (AZD1222)	NCT04324606	Produced strong neutralizing antibodies in 32/35 volunteers	Unspecified T-cell responses, up to 0.7% of cells in 43/43
Moderna (mRNA-1273)	NCT04283461	Strong level of neutralizing antibodies produced in 45/45 volunteers.	S-specific CD8+ responses, up to 0.2% of cells
Pfizer/Biontech (BNT162b1)	NCT04380701	Strong level of neutralizing antibodies produced in 48/48 volunteers.	RBD-specific CD8+ responses, up to 0.4% of cells in 29/36
Pfizer/Biontech (BNT162b1)	NCT04368728	Strong level of neutralizing antibodies produced in 36/36 volunteers.	No data
Gamelaya Research Institute-Sputnik V (Gam-COVID-Vac)	NCT04530396	Significant increases in neutralizing antibody titer after vaccination compared to placebo	Increased IFNγ production following stimulation of PBMCs with SARS-CoV-2 antigens
